# Coexistence with *Staphylococcus aureus* modulates the virulence and antibiotic resistance of *Pseudomonas aeruginosa*

**DOI:** 10.1186/s12941-025-00843-2

**Published:** 2026-01-28

**Authors:** Zeinab M. Helal, Soha Lotfy Elshaer, Mohammed El-Mowafy, Elsayed E. Habib

**Affiliations:** Department of Microbiology and Immunology, Faculty of Pharmacy, Mansoura University, Mansoura, 35516 Egypt

**Keywords:** *P. aeruginosa*, *S. aureus*, Co-existence, Antibiotic resistance, Pathogenicity

## Abstract

**Background:**

In this study, we were aimed to investigate the impact of co-cultures of different bacterial species on bacterial antibiotic resistance and virulence.

**Methods:**

The effect of co-cultures of *Pseudomonas aeruginosa* (Gram-negative bacteria) and *Staphylococcus aureus* (Gram-positive bacteria) on antibiotic resistance, virulence and biofilm formation in *P. aeruginosa* was examined in vitro in 14 mixtures. These mixtures were categorized into three groups: Standard category (including standard strains), naturally co-isolated category (co-isolated from the same patient) and random co-culture category (bacterial species from different patients). Additionally, the impact of the standard category on pathogenicity was assessed in vivo using mouse model. Intergroup comparisons were conducted using multiple *t*-test and comparisons between treated and untreated control isolates grown under the same conditions were made. Survival experiments were analyzed using Mantel-Cox log-rank test.

**Results:**

*P. aeruginosa* survival significantly increased in most of the co-culture mixtures when treated with meropenem (92.9%), ceftazidime (85.7%), cefepime (78.6%), gentamicin (78.6%) and ciprofloxacin (71.4%). Similarly, virulence factor production significantly increased in *P. aeruginosa* in most of the investigated co-cultures as follows: pyocyanin (71.4%), elastase (71.4%), protease (78.6%), hemolysin (71.4%), lecithinase (78.6%), gelatinase (63.6%) and biofilm (71.4%). At the molecular level, the relative expression of the tested virulence-encoding genes; *pelA*, *lasB* and *lasA* were significantly increased in at least 92.9% of the co-culture mixtures, especially in random ones, compared to their mono-culture, but with varying up-regulation degree (ranging from 1.5 to 96-fold increase).

**Conclusion:**

Finally, in vitro investigations for antibiotic resistance and virulence production clearly demonstrated a synergistic interaction between *P. aeruginosa* and *S. aureus* in the co-existence mixture. Compared to *P. aeruginosa* mono-cultures, the co-cultured strains exhibited enhanced resistance profiles and increased expression of key virulence factors, indicating that the simultaneous presence of both species promotes mutual adaptation and potentiation of pathogenic traits. Additionally, in vivo experiments confirmed that the co-infection with *S. aureus* significantly enhanced the pathogenicity of *P. aeruginosa*, as indicated by increased mortality rates and higher bacterial counts in lung tissues. Altogether, our results shed light on the impact of the co-existence of microbial species on bacterial virulence and antibiotic resistance.

**Supplementary Information:**

The online version contains supplementary material available at 10.1186/s12941-025-00843-2.

## Background

Interactions between microorganisms of mixed species within an infection site, could worsen the infection’s outcomes and increase the rate of morbidity and mortality among hospitalized patients [1]. This type of infection can target the respiratory tract, surgical wound or individuals suffering from diabetic foot and soft tissue infections, where *Pseudomonas aeruginosa* (*P. aeruginosa*) and *Staphylococcus aureus* (*S. aureus*) are the most prevalent and isolated pathogens [2, 3]. *P. aeruginosa* and *S. aureus* are frequently isolated from infected patients with cystic fibrosis (CF) [4]. The interaction between the two pathogens can affect their pathogenic behavior including triggering biofilm formation, virulence machinery and antimicrobial resistance, thus reducing the availability of treatment options during co-infections [5].

*P. aeruginosa* produces different virulence factors attributed to cell structure as flagellum, pilus and alginate, while others are secretory or exoenzymatic like pyocyanin, hemolysin and exotoxin [6]. In addition, it produces a wide range of extracellular proteases that help the pathogen during infection. Among them LasA, LasB elastase and alkaline protease are the most secreted under the regulation of quorum sensing (QS) [7]. Biofilm matrix formation by *P. aeruginosa* helps the bacterial cell withstand various environmental stresses including antibiotics and be guarded from the host immune defenses, ultimately worsen the infection state [8]. *S. aureus* is a significant human pathogen known for secreting a broad array of virulence factors including proteases, staphylokinase and coagulase that play important roles in its pathogenicity and antibiotic resistance. These factors help *S. aureus* pass through the skin and mucous membrane, leading to infection proliferation. These infections range from minor skin conditions to severe systemic diseases [9]. Furthermore, *S. aureus* secretes exotoxins, hemolysin, superantigens and Panton-Valentine leukocidin that facilitate immune evasion by disrupting host immune responses [9].

Healing of wounds infected with both *S. aureus* and *P. aeruginosa* takes much more time than those infected with either organism [10]. Several studies have been performed to determine whether co-infection between *P. aeruginosa* and *S. aureus* represents a transitional phase between infections caused by each pathogen alone or reflects a stable, permanent co-existence state [11]. It is important to consider the polymicrobial interactions between different microorganisms, as these interactions play significant roles in the disease’s pathogenesis and may influence the overall clinical outcomes. Identifying and analyzing these factors can greatly enhance our ability to manage and treat the disease more effectively [1]. The aim of this study was to understand the interaction (competition or co-existence) between different *P. aeruginosa* and *S. aureus* isolates and how this interaction affects *P. aeruginosa* resistance to different antibiotics and *P. aeruginosa* virulence factor production.

## Methods

### Bacterial strains and growth conditions

A total of 67 isolates were collected from different clinical sources. *P. aeruginosa* and *S. aureus* isolates were obtained either from different clinical samples or simultaneously from the same patient sample. Inclusion criteria involved patients with a specific infection e.g., a wound infection, pus, and diabetic foot, while exclusion criteria included start of antibiotic treatment and coinfections with other pathogens other than our target ones (*P. aeruginosa* and *S. aureus*). All specimens were obtained from different Mansoura University Hospitals and Clinics after approval from the ethical research committee, Faculty of Pharmacy, Mansoura University, following the medical research requirements in the usage, handling and care of human subjects (Ethical code:2025-97.). The standard strains *P. aeruginosa* PAO1 and *S. aureus* Newman were also included throughout the study. *P. aeruginosa* and *S. aureus* were recovered using cetrimide-supplemented agar (CA, Oxoid, Basingstoke, UK) and mannitol salt agar (MSA, Bio-life Italiana S.r.l. Milan, Italy), respectively and were further identified based on the standard microbiological recommendations [12]. All purified isolates were cryopreserved at − 80 °C as glycerol stocks (25%).

### Antibiotic sensitivity pattern

The antimicrobial susceptibility of the purified *P. aeruginosa* and *S. aureus* isolates was assessed by the Kirby-Bauer disc diffusion method [13], according to Clinical Laboratory Standard Institute guidelines (CLSI, 2021) [14], where we selected a representative member for each class of antibiotic suggested by the CLSI 2021. Pure bacterial colonies of *P. aeruginosa* and *S. aureus* in Muller-Hinton broth (MHB, Oxoid, Basingstoke, UK) medium were adjusted to an equal 0.5 McFarland, followed by streaking on Muller-Hinton agar plates. Different antimicrobial discs (Oxoid, Basingstoke, UK) included; cefepime (FEP,30 µg), amoxicillin/clavulanic acid (AMC, 20/10 µg), ceftazidime (CAZ,30 µg), aztreonam (ATM, 30 µg), ciprofloxacin (CIP, 5 µg), gentamicin (CN, 10 µg) and meropenem (MEM, 10 µg) for *P. aeruginosa* isolates. While cefepime (FEP,30 µg), amoxicillin/clavulanic acid (AMC, 20/10 µg), gentamicin (CN, 10 µg), ciprofloxacin (CIP,5 µg), azithromycin (AZM,15 µg) and doxycycline (DO, 30 µg) were used for *S. aureus* isolates. After overnight incubation of the plates at 37 °C, the inhibition zones were measured and the results were interpreted according to CLSI [14].

### Growth competition assay

The growth competition assay, via drop plate method, was performed to investigate how co-cultured *S. aureus* and *P. aeruginosa*, isolated from the same or different patients, co-exist or compete with each other [15]. Overnight cultures of both *P. aeruginosa* and *S. aureus* in Tryptic soy broth (TSB, Oxoid, Basingstoke, UK) were adjusted to 0.5 McFarland and further sub-cultured as co-cultures (1:1 mixed ratio) or as mono-cultures (1:1 mixed ratio with sterile TSB medium) and incubated with shaking at 37 °C for 24 h. One hundred µL were taken at 2 h intervals for 8 h, ten-fold serially diluted and streaked onto CA medium for enumeration of *P. aeruginosa* colonies (CA plates allow the growth of *P. aeruginosa* but not *S. aureus*). The experiment was carried out in triplicate to ensure reproducibility and the average number of colonies was obtained. Colony-forming unit (CFU) /mL on CA plates were determined for streaked mono-culture of *P. aeruginosa* or its co-culture with *S. aureus*, then the fold change (FC) in the growth of *P. aeruginosa* was calculated as follows: FC = CFU/mL of co-cultured *P. aeruginosa* / CFU/mL of mono-culture of the corresponding *P. aeruginosa* isolate at each time interval [4, 16]. The co-cultured mixtures showing competitive interaction were excluded from further experiments in this work.

### The effect of coculturing of *S. aureus* and *P. aeruginosa* on antibiotic susceptibility of *P. aeruginosa*

The minimum inhibitory concentrations (MIC) of cefepime, ceftazidime, gentamicin, ciprofloxacin and meropenem were firstly determined for *P. aeruginosa* mono-culture in MHB via the microbroth dilution method, using a 96-well plate with an initial inoculum of 10^6^ CFU/mL [14]. MIC was determined as the lowest concentration showing no visible growth at OD_600nm_. After that, the overnight mono-cultures of either *P. aeruginosa* or *S. aureus* in MHB were adjusted to 10^6^ CFU/mL and further sub-cultured as co-culture (1:1 mixed ratio) or as mono-cultures (1:1 mixed ratio with MHB) in the presence of 0.5x MIC of the tested antibiotics. After incubation of plates at 37 °C, 5 µL of each co-culture and its corresponding mono-cultures, challenged with the 0.5x MIC of each antibiotic, were plated on CA for enumeration of *P. aeruginosa* colonies. The percentage of bacterial survival after antibiotic treatment was determined by dividing the number of *P. aeruginosa* colonies (in mono- or co-culture) after antibiotic treatment by that without antibiotic treatment. Afterwards, the fold change in *P. aeruginosa* survival rate was calculated as follows: FC= % of survival of co-cultured *P. aeruginosa* after antibiotic treatment / % survival of *P. aeruginosa* mono-culture after antibiotic treatment [17].

### The effect of coculturing *S. aureus* and *P. aeruginosa* on virulence factors of *P. aeruginosa*

The impact of the co-existence of *P. aeruginosa* and *S. aureus* on the virulence of *P. aeruginosa* including the production of: pyocyanin, elastase, total proteases, hemolysin, lecithinase and gelatinase was examined. Individual cultures of *P. aeruginosa* and *S. aureus* were cultivated at 37 °C. The overnight cultures were adjusted to 0.5 McFarland and further sub-cultured as co-cultures (1:1 mixed ratio) or as mono-cultures (1:1 mixed ratio with sterile Luria-Bertani (LB, Oxoid, Basingstoke, UK) broth medium and incubated at 37 °C (16–18 h) followed by centrifugation. The supernatants of mono-cultures or co-cultures were stored at -80 °C, after sterile filtration with 0.22 µM filters, until further assay of the virulence factors of *P. aeruginosa*.

Regarding the assay of elastase and pyocyanin (produced only by *P. aeruginosa*), the supernatants of mono-cultures were compared with those of co-cultures. The percentage change in elastase or pyocyanin production in *P. aeruginosa* after co-culturing was calculated as follows: 100* Production of virulence factor of *P. aeruginosa* in co-culture/ Production of virulence factor of *P. aeruginosa* in mono-culture. On the other hand, in the case of virulence factors produced by both *P. aeruginosa* and *S. aureus* (total proteases, hemolysin, lecithinase, and gelatinase), the mixtures of supernatants of mono-cultures of both bacterial species (1:1) were compared to those of the corresponding co-cultures mixed with sterile LB medium (1:1). The percent of change in the virulence factors production in *P. aeruginosa* after co-culturing was calculated as follows: 100* Production of the virulence factor in co-cultured supernatant/ Production of virulence factor in supernatant mixtures of their corresponding *P. aeruginosa* and *S. aureus* mono-cultures.

For pyocyanin quantification, the supernatant was extracted with chloroform, and the organic layer containing pyocyanin was extracted with 0.2 N HCL (El-Nasr pharmaceutical chemicals Co. Cairo, Egypt), then the pink color was measured at OD_520nm_ [18].

Elastase activity was measured in the supernatant by the elastin Congo red (ECR, Sigma Aldrich, St. Louis, MO, USA) method and the developed color was measured at OD_495nm_ [19].

Measurement of total proteases was done using a skimmed milk procedure, where the increase in the proteolytic activity of the dual-culture was estimated through a decrease in OD_600nm_ absorbance reading after comparison with a negative control that comprised LB medium instead of supernatant [20].

The hemolysis activity was measured according to [21], where the supernatants were incubated with 2% RBCs suspended in hemolysin buffer for 2 h at 37 °C. LB medium and 1% SDS were used instead of the supernatant as negative and positive control, respectively. Then, the percentage of RBCs lysis was calculated as follows: 100* (OD_600nm_ of tested supernatant - negative control)/ (OD_600nm_ of positive - negative control).

Lecithinase activity was investigated in the supernatant after mixing with 10% egg yolk suspension in sterile 96-well microtiter plate, followed by incubation for 72 h at 37 °C. LB broth was used as negative control instead of bacterial supernatant. The absorbance of the white-colored solution was measured at 600 nm, where higher absorbance indicated higher lecithinase production [22].

Gelatinase production was measured using gelatin agar plates according to [23], where 5 µL of each supernatant was spotted on gelatin plates and incubated overnight at 37 °C. Afterwards, the plates were flooded with saturated ammonium sulfate solution. Gelatin hydrolysis was indicated by clear zones around the applied spots in gelatin media. Among the 14 investigated co-culture mixtures, only mixtures in which *P. aeruginosa* mono-cultures producing gelatinase were included in the gelatinase assay.

Quantitative biofilm formation was assessed using the polystyrene microtiter plate adsorption method [24]. Overnight mono-cultures of *P. aeruginosa* and *S. aureus*, propagated in TSB, were diluted to 0.5 McFarland and cultivated as co-culture (1:1 mixed ratio) or as mono-culture. The wells were inoculated with 200 µL of each mono-or co-cultured suspension, in addition to negative control wells that were filled with 200 µL of TSB. After incubation overnight at 37 °C, non-adherent planktonic growth was gently aspirated and rejected. The attached biofilm in each well was washed twice with sterile saline, fixed with methanol (El-Nasr pharmaceutical chemicals Co. Cairo, Egypt) (200 µL) for 20 min and stained with 1% w/v crystal violet (200 µL) for 15 min. The plates were dried after rinsing the excess crystal violet, while the adhered stained biofilm was solubilized by 33% v/v glacial acetic acid (El-Nasr pharmaceutical chemicals Co. Cairo, Egypt) (200 µL/ well).

To study the effect of the co-culture of *S. aureus*/ *P. aeruginosa* on biofilm formation by the two pathogenic bacteria, the measured OD_T_ of the solubilized biofilm in the case of co-culture mixtures or the mixture of the solubilized biofilm of the corresponding individual mono-cultures were categorized according to biofilm formation into non-adherent (OD_T_ ≤ OD_C_), weak-adherent (ODc < OD_T_≤ 2ODc), moderate-adherent (2ODc < OD_T_ ≤ 4ODc, ) and strong-adherent (4ODc < OD_T_), where OD_C,_ a cutoff value, was estimated as three standard deviations (SD) above the mean OD_570 nm_ of the negative control [25]. The percentage change in biofilm formation for co-culture in comparison with mixtures of corresponding individual mono-cultures was calculated as follows: 100* OD_T_ of the solubilized biofilm of co-culture / OD_T_ of the mixture of the solubilized biofilm of the corresponding individual mono-cultures of both bacterial species.

### Real-time quantitative PCR

RT-PCR was used to measure the effect of the co-existence of *S. aureus* on the expression of some virulence-encoding genes in *P. aeruginosa* including; *pelA*, *lasB* and *lasA*. It should be mentioned that we performed PCR screening for 11 genes; including secretory virulence gene (*lasA*,* lasB*,* exoS*,* exoY*,* toxA* and *PIcH*), biofilm gene (*pelA*) and quorum sensing genes *(lasI*, *lasR*, *RhIR* and *Rhll*). However, only the *lasA*, *lasB* and *pelA* genes were harbored by all the investigated isolates (Data not shown). Therefore, the last genes were further adopted in the RT-PCR experiments. Individual cultures of *P. aeruginosa* and *S. aureus* were cultivated at 37 °C. The overnight cultures were adjusted to 0.5 McFarland and further incubated as co-cultures (1:1 mixed ratio) or as mono-cultures. Afterwards, 100 µL of both co-culture and corresponding *P. aeruginosa* mono-culture were sub-cultured separately in 10 mL LB media, incubated with shaking till the mid exponential phase (OD_600_ = 0.5–0.6) was reached [26]. Cells of mono-and co-cultures were obtained by centrifugation at 4 ℃ for 20 min at 8000 rpm and the total RNA was extracted using TRI reagent (Sigma-Aldrich, St. Louis, MO, USA) based on the manufacturer’s guidelines. DNA contamination was removed using DNase enzyme (Thermo Scientific™, Waltham, Massachusetts, USA) according to the manufacturer’s guidelines. RNA concentration was measured by NanoDrop (Wilmington, Delaware, USA). The cDNA synthesis was performed by Thermo Scientific revertAid kit (Thermo Scientific™, Waltham, Massachusetts, USA). RT-PCR was done by Hera plus sybr green master mix (HERAPLUS SYBR Green, Willowfort, Nottingham, UK) along with primers and conditions listed in Table 1 and the reaction was performed in Rotor-Gene Q thermocycler (Qiagen, Hilden, Germany). The relative expression levels of *P. aeruginosa* virulence genes were normalized to the expression levels of *ropD* housekeeping gene in accordance with the 2^−∆∆CT^ [27]. Finally, the impact of *S. aureus* / *P. aeruginosa* co-culture on gene expression (*pelA*, *lasB* and *lasA*) of *P. aeruginosa* was determined after considering the expression of each gene in *P. aeruginosa* mono-culture as control.

**Table 1:** List of primers used in this study

Gene	Primer name	Sequence (5′→3′)	Annealing temperature (°C)	Amplicon size (bp)	Reference
*ropD*	*ropD* F	CGAACTGCTTGCCGACTT	56	131	[28]
	*ropD* R	GCGAGAGCCTCAAGGATAC			
*pelA*	*pelA* F	AAGAACGGATGGCTGAAGG	58	148	[28]
	*pelA* R	TTCCTCACCTCGGTCTCG			
*lasB*	*lasB* F	GGTAGAACGCACGGTTGT	56	165	[28]
	*lasB* R	GGCAAGAACGACTTCCTGAT			
*lasA*	*lasA* F	CTC GCC GTT CCT CTT CGT CT	58	86	[29]
	*lasA* R	GCC ATC GTC ATG GGC ATT GG			

F or R in the primer name refers to forward and reverse primer, respectively

### In vivo assay

#### Mice

Female BALB/c mice (Conventional), aged six to eight weeks and weighing between 19 and 20 g, were utilized in this study. BALB/c mice are well recognized for their high immunological responsiveness and sensitivity, making them one of the most widely used strains in immunological and infectious disease research. Male BALB/c mice are typically more aggressive and exhibit strong territorial behavior, which makes group housing challenging and may induce stress that interferes with immune parameters. In contrast, female BALB/c mice demonstrate greater consistency in experimental outcomes, largely attributed to differences in hormonal physiology and immune regulation mechanisms [30–32].

Mice were provided with food (standard rodent chow), water and 12-h light/dark cycles at a temperature of 22 ± 2 °C. Mice were placed in well ventilated cages for each group and monitored daily for distress or illness. Clinical assessment included fur condition, activity level, body weight and response to handling. A scoring system (0–3 scale for each parameter) was applied and humane endpoints were defined as > 20% weight loss, severe lethargy or inability to eat or drink. At the end of the experiment or upon reaching humane endpoints, mice were anesthetized with isoflurane (Universal Pharma Co, Cairo, Egypt) and euthanized by cervical dislocation to ensure rapid and humane death [33].

### Determination of non-lethal dose of each bacterial species

The bacterial non-lethal dose of *P. aeruginosa* PAO1 and *S. aureus* Newman strain was determined, where different groups of mice were subjected to intranasal administration of different bacterial inocula (10^9^, 10^7^ and 10^5^ CFU) in 20 µL of phosphate buffered saline (PBS). Animals were monitored daily for survival and weight variation [34–36].

### In vivo co-cultures

Six different groups of mice were used. Five mice were randomly selected to be included in each group of the experiment. After anesthesia, mice groups were subjected to single intranasal administration of 20 µL (containing non-lethal inoculum 10^5^ CFU) of each of the following; *P. aeruginosa* PAO1 strain only (PA group), *S. aureus* Newman strain only (SA group), *P. aeruginosa* PAO1 strain followed by intranasal administration of *S. aureus* Newman strain after three days (PA-SA group), *S. aureus* Newman strain followed by *P. aeruginosa* PAO1 strain after three days (SA-PA group), both *P. aeruginosa* PAO1 strain and *S. aureus* Newman strain simultaneously (PA + SA group) and the non-infected group (NI group) which received sterile PBS only. Animals were monitored for one week for survival, weight and behavior changes. After a week, lungs were aseptically collected using sterile instruments and transferred immediately into pre-weighed sterile tubes containing 1 mL of sterile PBS, followed by homogenization using sterile tissue homogenizer, and the homogenized lung suspensions were serially diluted in PBS. Such dilutions were plated on CA plates and incubated at 37 °C for 18–24 h to determine viable bacterial counts of *P. aeruginosa* that were expressed as colony-forming units (CFU/g tissue) [34–36].

The primary endpoint of the study was bacterial burden in target organs (expressed as CFU/g) at predefined time points post-infection. Secondary endpoints included survival up to [one week], change in body weight, clinical score, and histopathological assessment. Humane endpoints were implemented to minimize animal suffering: animals were monitored twice daily and euthanized if they met any of the following criteria — ≥20% body weight loss, severe dyspnea (use of accessory respiratory muscles or cyanosis), inability to eat/drink for > 48 h, hypothermia, severe lethargy/unresponsiveness, or any clinical score meeting or exceeding the humane threshold [37, 38]. All procedures, care and handling of the animals were performed according to the institutional and national ethical guidelines. Additionally, the study was approved by the Animal Care and Use Committee, Mansoura university (MU-ACUC (PHARM.PHD.24.05.41).

### Histological evaluation

Histological evaluation was performed using single mice from each group. Lungs were collected, fixed in formalin and embedded in paraffin for histological sections (5 μm) after staining with Hematoxylin-Eosin, followed by microscopic observation [39].

### Statistical analysis

Excel (Microsoft Office) was used to calculate the average and SD of each experiment, which was conducted in triplicate. Graph Pad Prism software package (version 6.01) was used for all the statistical analyses. The multiple *t*-test was utilized for intergroup comparisons in addition to comparisons between treated and untreated control isolates cultivated under the same circumstances. Survival experiments were analyzed using the Mantel-Cox log-rank test; with *n* = 5/group, while non-parametric *t*-test was used in the case of bioburden assays.

## Results

### Design of co-culture mixtures and detection of growth interaction type

A total of 29 isolates of *P. aeruginosa* and 38 isolates of *S. aureus* were obtained from different clinical sources (Table S1 supplementary material). The *P. aeruginosa* isolates, were given the codes; P1-29, while *S. aureus* were given the codes S1-38. The antibiotic resistance patterns for the *P. aeruginosa* and *S. aureus* isolates are demonstrated in Tables 2 and 3 supplementary material. The co-cultures of *P. aeruginosa* and *S. aureus* (16 mixtures) that were investigated in this study were classified into 3 categories; standard category, naturally co-isolated category (co-isolated from the same patient) and random co-cultures category (Table 2). It should be mentioned that the three naturally co-isolated mixtures were not subjected to repeated subculture to retain their original characters.

The most resistant and sensitive isolates of either *P. aeruginosa* or *S. aureus*, resistant and sensitive to most classes of antibiotics were selected for the design of co-culture categories. Such resistant isolates of *P. aeruginosa* included P1, P2, P6 and P7, while for *S. aureus* it comprised S4, S5, S6 and S7. On the other side, P3, P4 and P5 were selected as the most sensitive *P. aeruginosa* isolates, while S1, S2, S3 and S8 were the most sensitive *S. aureus* isolates (Tables S2 and S3 supplementary material).

**Table 2:** Design of co-cultures categories used in this study

Category	Co-culture composition		Code of the co-culture
	*P. aeruginosa*	*S. aureus*	
Standard	*P. aeruginosa* strain PAO1	*S. aureus* strain Newman	PAO1/Newman
	*P. aeruginosa* strain PAO1	Randomly selected sensitive isolate (Ss2)	PAO1/Ss2
	*P. aeruginosa* strain PAO1	Randomly selected resistant isolate (Sr4)	PAO1/Sr4
	Randomly selected sensitive isolate (Ps4)	*S. aureus* strain Newman	Ps4/Newman
	Randomly selected resistant isolate (Pr7)	*S. aureus* strain Newman	Pr7/Newman
Natural co-isolated	P8	S9	P8/S9
	P9	S10	P9/S10
	P10	S11	P10/S11
Random designed	Resistant isolate (Pr2)	Sensitive isolate (Ss3)	Pr2/Ss3
	Sensitive isolate (Ps3)	Sensitive isolate (Ss3)	Ps3/Ss3
	Resistant isolate (Pr1)	Resistant isolate (Sr5)	Pr1/Sr5
	Sensitive isolate (Ps5)	Resistant isolate (Sr5)	Ps5/Sr5
	Sensitive isolate (Ps5)	Resistant isolate (Sr7)	Ps5/Sr7
	Sensitive isolate (Ps5)	Sensitive isolate (Ss8)	Ps5/Ss8
	Resistant isolate (Pr6)	Sensitive isolate (Ss1)	Pr6/Ss1
	Resistant isolate (Pr6)	Resistant isolate (Sr6)	Pr6/Sr6

Pr: Resistant *P. aeruginosa* isolate, Ps: Sensitive *P. aeruginosa* isolate, Sr: Resistant *S. aureus* isolate, Ss: Sensitive *S. aureus* isolate

All co-cultures exhibited co-existence interaction except Ps5/Sr7 and Ps5/Ss8 mixtures which showed antagonistic interaction. The FCs in the growth of *P. aeruginosa* strains after 6 and 8 h cultivation in co-cultures are demonstrated in Table 3.

**Table 3:** FCs in the growth of *P. aeruginosa* co-culture after 6 and 8 h cultivation

Category	Co-culture code	FC in growth of *P. aeruginosa* after	
		6 h	8 h
Standard	PAO1/Newman	600	1153
	PAO1/Ss2	500	727
	PAO1/Sr4	933	1800
	Ps4/Newman	1142	942
	Pr7/Newman	727.3	500
Naturally co-isolated	P8/S9	1200	2200
	P9/S10	1026.3	1119
	P10/S11	1055.6	1280
Random designed	Pr2/Ss3	900	980
	Ps3/Ss3	2400	1093
	Pr1/Sr5	824	843.7
	Ps5/Sr5	3333	2208
	Pr6/Ss1	857	1889
	Pr6/Sr6	857	2222
Competitive category	Ps5/Sr7	0.007	0.004
	Ps5/Ss8	0.009	0.006

### The effect of co-culture with *S. aureus* on *P. aeruginosa* antibiotic susceptibility

**Fig. 1:**
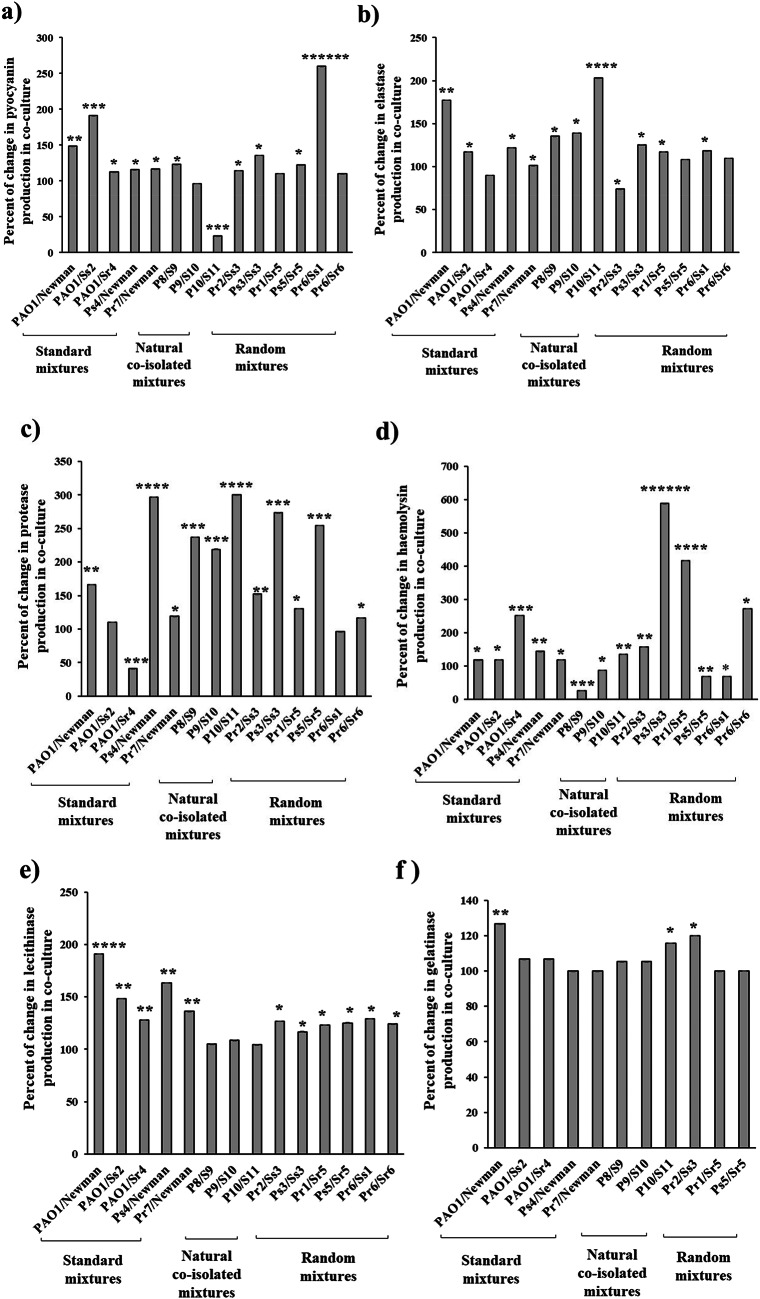
Fold change of survival rates of *P. aeruginosa* in co-cultures in presence of 0.5x MIC of antibiotics. **a** Cefepime, **b** Ceftazidime, **c** Gentamicin, **d** Ciprofloxacin and **e** Meropenem. * *P* < 0.05, ** *P* < 0.01, ***P < 0.005, ****P < 0.001, *****P < 0.0005 and ****** *P* < 0.0001

Most of *P. aeruginosa* in co-culture mixtures showed an increase in survival rate in the presence of 0.5x MIC of the investigated antibiotics. The highest significant increase in the survival rate of *P. aeruginosa* was obtained with meropenem in 13 co-culture mixtures (92.9%), followed by ceftazidime (12 mixtures, 85.7%), cefepime (11 mixtures, 78.6%) and gentamicin (11 mixtures, 78.6%) (Fig. 1).

The highest increase in FC of survival rates in *P. aeruginosa* was observed with cefepime (2.2*10^7^ FC, Pr1/Sr5 mixture) (Fig. 1a), followed by ciprofloxacin (518075 FC, P9/S10 mixture) (Fig. 1d) and ceftazidime (1*10^5^ FC, Pr7/Newman mixture) (Fig. 1b).

### The effect of co-culture with *S. aureus* on *P. aeruginosa* virulence factors production

**Fig. 2:**
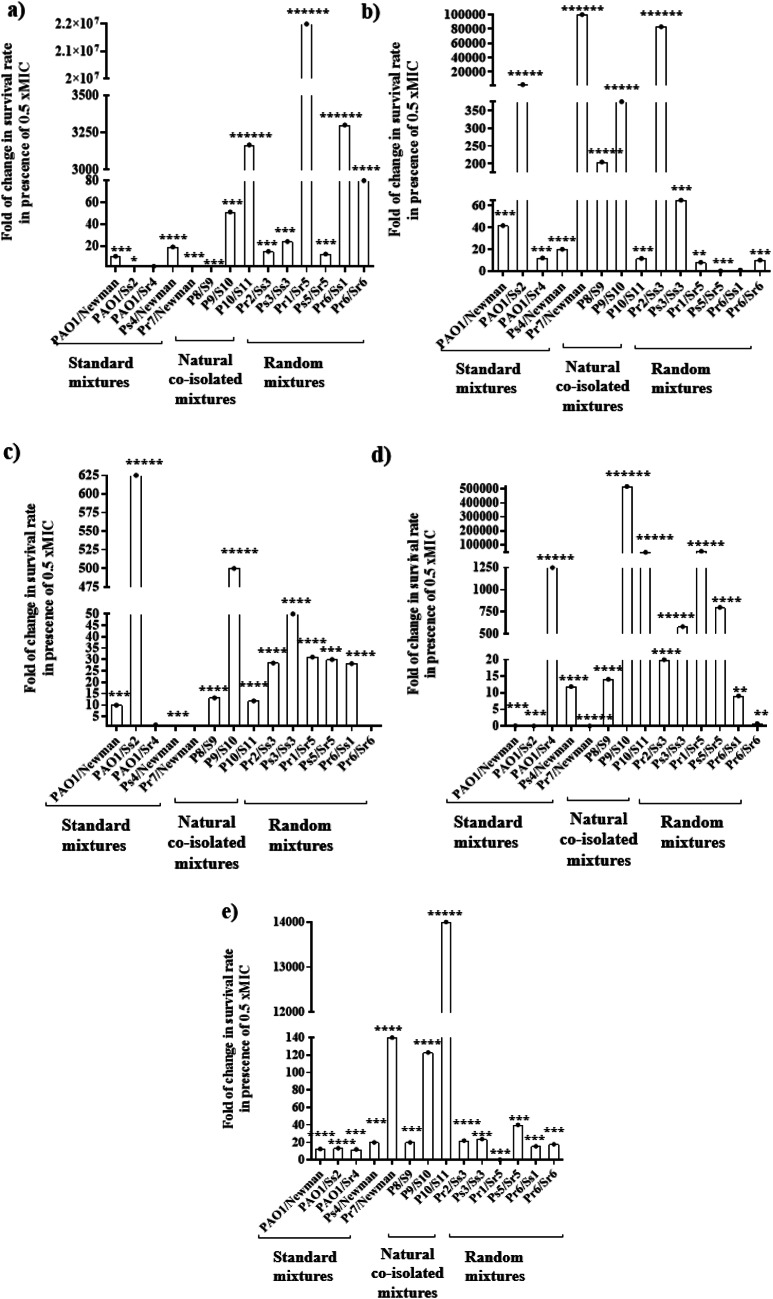
Effect of *S. aureus* co-culture on virulence factors’ production in *P. aeruginosa*. **a** Pyocyanin, **b** Elastase, **c** Protease, **d** Hemolysin, **e** Lecithinase, **f** Gelatinase. The SD of the three independent experiments is illustrated by error bars. * *P* < 0.05, ** *P* < 0.01, ***P < 0.005, ****P < 0.001 and ****** *P* < 0.0001

A significant increase in pyocyanin production of *P. aeruginosa* was observed in ten co-culture mixtures (71.4%, *p* = 0.008) compared to pyocyanin production in their corresponding mono-culture of *P. aeruginosa*, where the Pr6/Ss1 mixture was the highest in increasing pyocyanin production (260%), while only the mixture P10/S11 demonstrate a significant decrease in pyocyanin production (Fig. 2a).

Regarding elastase production, a significant increase in elastase production was obtained in ten co-culture mixtures (71.4%, *p* = 0.036) including all the naturally co-isolated ones, compared to elastase production by *P. aeruginosa* mono-culture, the mixture P10/S11 was the highest in increasing elastase production by 200% (Fig. 2b), while only Pr2/Ss3 showed significant decrease in elastase production.

Concerning proteolytic activities, a significant increase was produced by 11 co-culture mixtures (78.6%, *p* = 0.007) including all naturally co-isolated ones, compared to their controls, where the mixtures P10/S11, Ps4/ Newman and Ps3/Ss3 were the highest for increasing proteolytic activities by 300, 297% and 273.3%, respectively. On the other hand, the two mixtures PAO1/Sr4 and Pr6/Ss1 showed a decrease in proteolytic activities (Fig. 2c).

A significant increase in hemolysin production was observed in ten co-culture mixtures (71.4%), including all the standard ones. The randomly designed mixture, Ps3/Ss3 was the highest in increasing hemolysin production by 588% (Fig. 2d).

Regarding lecithinase production, a significant increase was produced among 11 co-culture mixtures (78.6%, *p* < 0.0002), including all standard and randomly designed ones. The standard mixtures, PAO1/ Newman, Ps4/ Newman and PAO1/Ss2 were the highest to increase lecithinase activities by 191%, 163.6% and 148%, respectively. No significant increase was demonstrated among the three natural co-isolated mixtures (Fig. 2e).

For gelatinase production activities, the three co-culture mixtures; Ps3/Ss3, Pr6/Ss1 and Pr6/Sr6 were not investigated because *P. aeruginosa* isolates in these mixtures do not produce gelatinase. Among 11 investigated co-culture mixtures, only 7 mixtures (63.7%) demonstrated an increase in gelatinase production. The PAO1/Newman mixture was the highest in increasing gelatinase production activities by 126.7% (Fig. 2f).

**Fig. 3:**
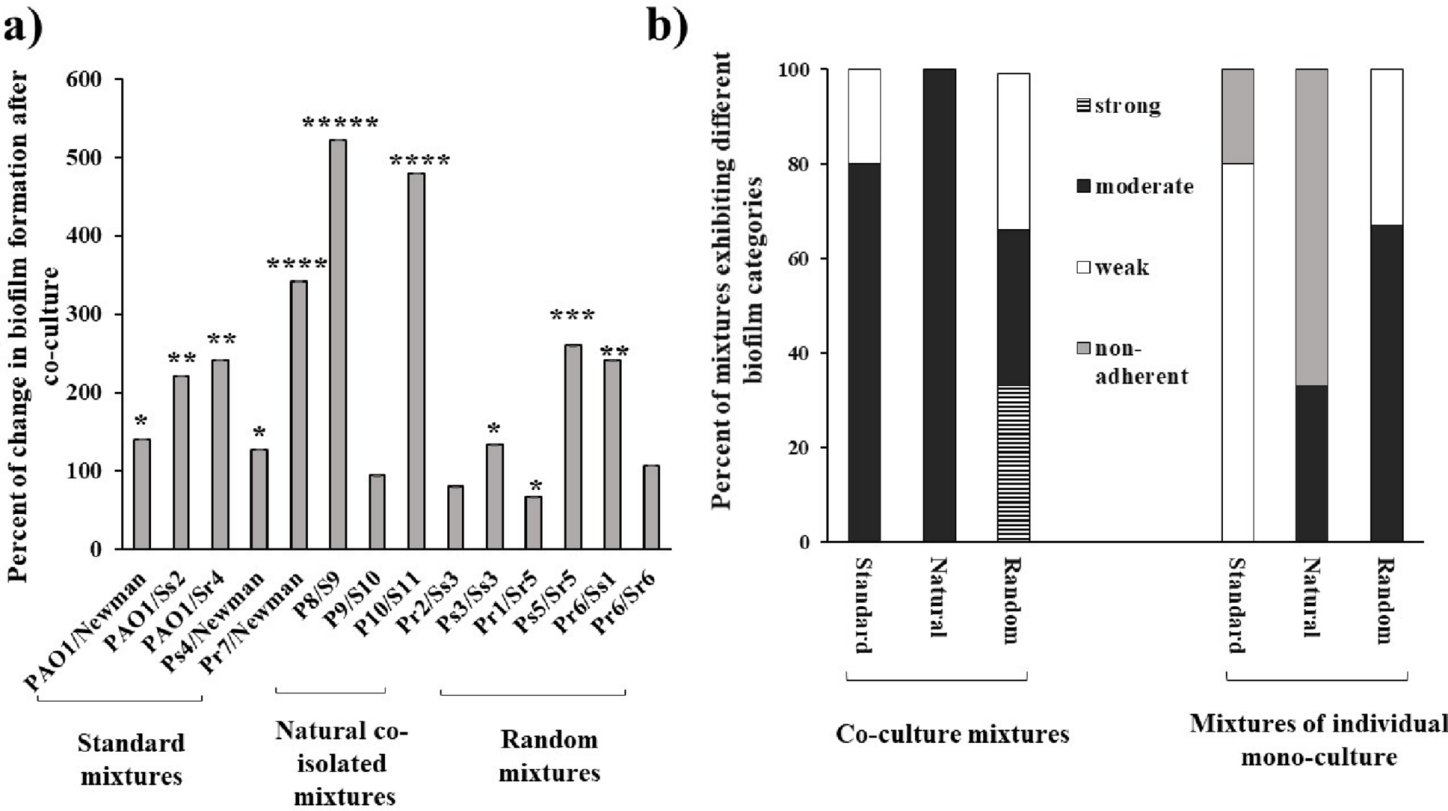
Effect of *S. aureus* co-culture on biofilm production in *P. aeruginosa.* (a) Percent of change in biofilm formation after co-culture, (b) Percent of mixtures exhibiting different biofilm categories. SD of the three independent experiments is illustrated by error bars (* *P* < 0.05, ** *P* < 0.01, ***P < 0.005, ****P < 0.001 and *****P < 0.0005)

Pertaining biofilm formation by *P. aeruginosa*, a significant increase was produced by ten of co-culture mixtures (71.4%, *p* = 0.006), where the P8/S9, the naturally co-isolated mixture, was the highest biofilm-forming mixture (522%) (Fig. 3a).

Among the five standard mixtures, four co-cultures (80%) turned to be moderate biofilm producers, whereas their mixtures of corresponding individual mono-cultures were weak biofilm and non-biofilm producers (Fig. 3b). Additionally, two mixtures of the naturally co-isolated ones (66.7%), P8/S9 and P10/S11, demonstrated moderate biofilm production compared to their mixtures of corresponding individual mono-cultures that were non-biofilm producers. Furthermore, Ps5/Sr5 and Pr6/Ss1, the two random selected mixtures, turned to produce strong biofilm adhering activity, where their mixtures of corresponding individual mono-cultures were classified as moderate-adherent (Fig. 3b) (Table S4 supplementary material).

### RT-PCR analysis

According to the 2^−∆∆CT^ analysis method, the expression level of *pelA*, *lasB* and *lasA* virulence genes in either *P. aeruginosa* mono-cultures or those co-cultured was quantified and further relatively normalized to the expression level of *ropD* housekeeping gene expressed in the same mono-or co-culture. This normalized value of each tested gene expression in co-culture relative to mono-culture was used to obtain the fold change.

**Fig. 4:**
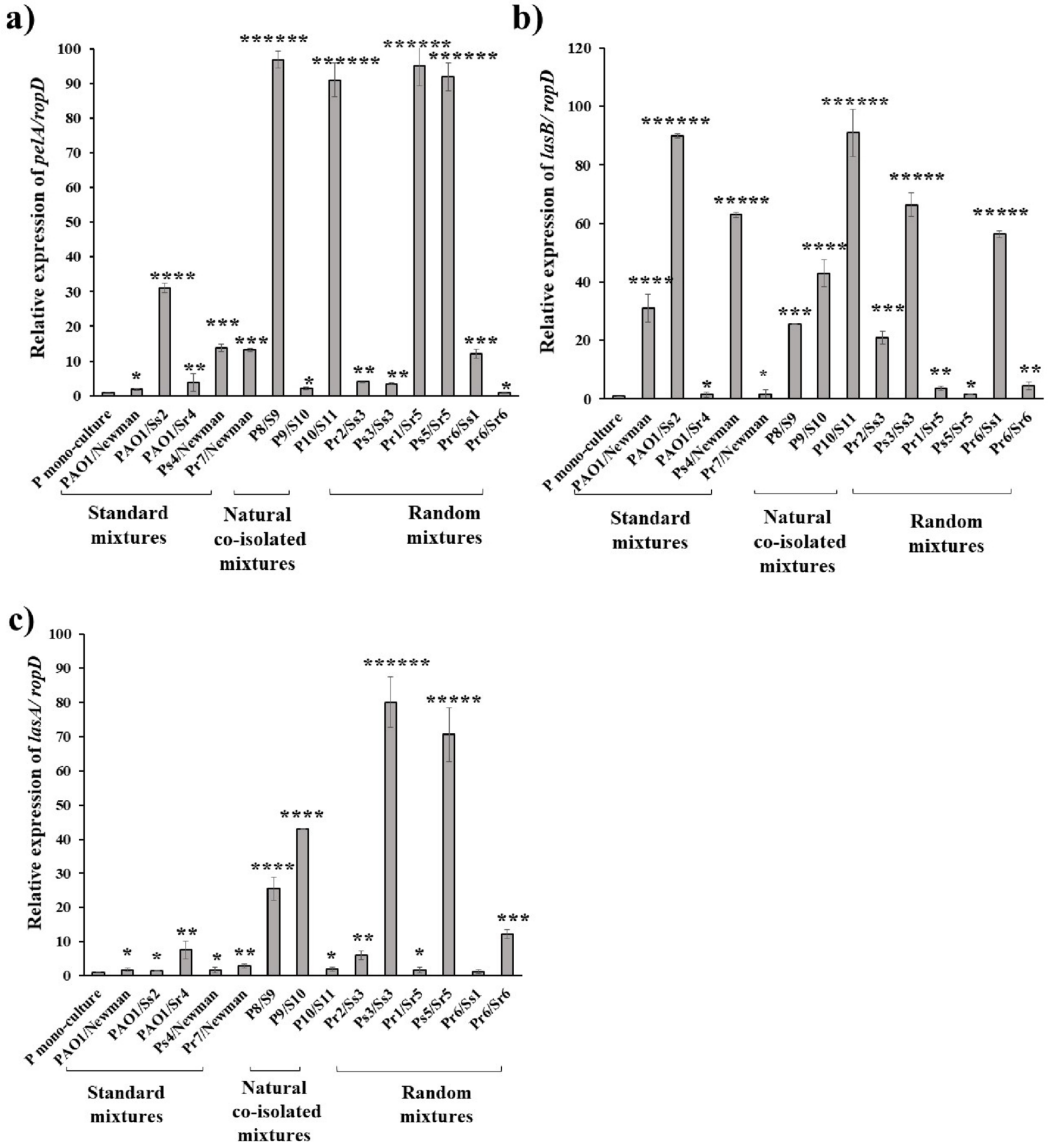
Effect of *S. aureus* co-culture on relative expression of virulence genes in *P. aeruginosa*. **a** Relative *pelA* expression, **b** Relative *lasB* expression, **c** Relative *lasA* expression. SD of the three independent experiments is illustrated by error bars (* *P* < 0.05, ** *P* < 0.01, ***P < 0.005, ****P < 0.001, *****P < 0.0005 and ****** *P* < 0.0001)

The data revealed that *pelA* was significantly up-regulated in 13 mixtures (92.9%, *p* = 0.01), including all the standard and naturally co-isolated mixtures. Tremendous increase (*p* < 0.0001) in *pelA* expression was shown in two natural co-isolated mixtures (P8/S9 and P10/S11) and two random mixtures (Pr1/Sr5 and Ps5/Sr5) by 96-, 91-, 95- and 92-fold, respectively, compared to their corresponding *P. aeruginosa* mono-culture. While, *pelA* expression was significantly (*p* < 0.05) down-regulated by 22% in only the random Pr6/Sr6 mixture, compared to Pr6 isolate (Fig. 4a).

Regarding *lasB* gene, it was significantly up-regulated among all the co-culture mixtures, but with varying degree (*p* = 0.001, Fig. 4b). Compared to *P. aeruginosa* mono-culture, the fold change in *lasB* expression started from a slight increase (by 1.5–4.5-fold) in PAO1/Sr4, Pr7/Newman, Pr1/Sr5, Ps5/Sr5 and Pr6/Sr6 till very noticeable increase (by > 50–91-fold) in PAO1/Ss2, Ps4/Newman, P10/S11, Ps3/Ss3 and Pr6/Ss1 (*p* < 0.0001).

Additionally, *lasA* gene was significantly up-regulated in 13 co-culture mixtures (92.9%, *p* = 0.03), since the highest relative expression was in random mixtures, Ps3/Ss3 and Ps5/Sr5, by 80- and 71-fold, respectively. A slight increase (18%), with no significant difference, in *lasA* expression was observed in only Pr6/Sr1 mixture, comparing with *P. aeruginosa* mono-culture (Fig. 4c).

### In vivo assay

#### Determination of non-lethal dose of each bacterial species

The inoculum 10^9^ CFU/ mL cause death within 24 h for all mice in the investigated group, while the inoculum 10^7^ CFU/ mL affected the mice severely causing the mice to stop eating, leading to severe weight loss, followed by mice death after 48 h. The 10^5^ CFU/ mL inoculum was associated with clinical signs consistent with systemic infection and respiratory distress, but the mice recovered from the symptoms after 3 days of infection. Therefore, the inoculum 10^5^ CFU/ mL was selected in further experiments.

### Co-infection with *S. aureus* Newman strain increased morbidity and mortality in *P. aeruginosa* PAO1 strain-infected mice

**Fig. 5:**
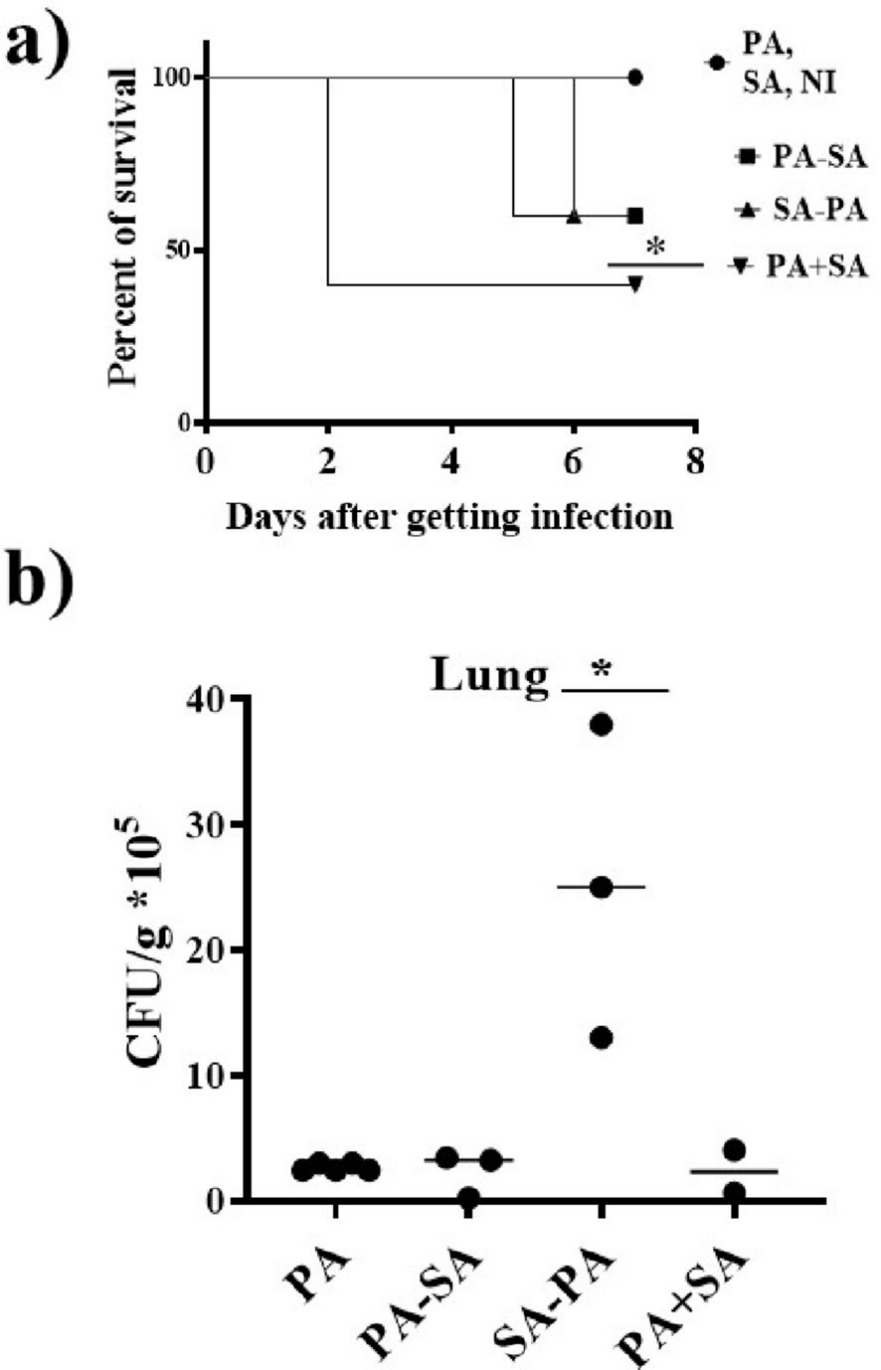
Survival rates and bacterial counts in lungs of mice models after co-infection with *P. aeruginosa* PAO1 and *S. aureus* Newman strains. **a** Cumulative survival rate of different mice groups and **b** *P. aeruginosa* PAO1 strain burdens in lungs of infected mice groups. Dots represent CFU in the lung of individual mice, and horizontal lines represent median values. PA and SA group: Means mice got infection with *P. aeruginosa* PAO1 strain and *S. aureus* Newman strain, respectively, NI group: non-infected mice that got sterile PBS only, PA-SA group: Mice infected with *P. aeruginosa* PAO1 strain followed *S. aureus* Newman strain three days a part, SA-PA group: Mice infected with *S. aureus* Newman strain followed by *P. aeruginosa* PAO1 strain three days apart, PA + SA group: Mice infected with *P. aeruginosa* PAO1 strain and *S. aureus* Newman strain simultaneously (* *P* < 0.05)

By tracking mice death, it was found that *S. aureus* Newman strain increased the death in mice groups that were co-infected by *P. aeruginosa* PAO1 and *S. aureus* Newman strains, where three dead mice were found in the PA + SA group by the second day of infection. PA-SA group and SA-PA group have the same rate of mortality (40%); however, the SA-PA group was associated with an earlier mortality rate at the second day in comparison with the PA-SA group that was at the third day. On the other side, all mice of control groups PA, SA and NI were still alive till the end of the experiment (Fig. 5a).

Regarding *P. aeruginosa* PAO1 strain burdens in the lung of the mice that were still alive after the 7 days of the experiment, we observed that, mice that were co-infected by *P. aeruginosa* PAO1 strain and *S. aureus* Newman strain simultaneously did not significantly affect the *P. aeruginosa* PAO1 strain count in the investigated lungs in comparison with the PA group. On the other side, pre-infection with *S. aureus* Newman strain, in SA-PA group, significantly increased *P. aeruginosa* PAO1 burdens in lungs of the investigated mice (*P* < 0.05, Fig. 5b).

### *S. aureus* Newman increased the inflammation in the lung of *P. aeruginosa* PAO1-infected mice

**Fig. 6:**
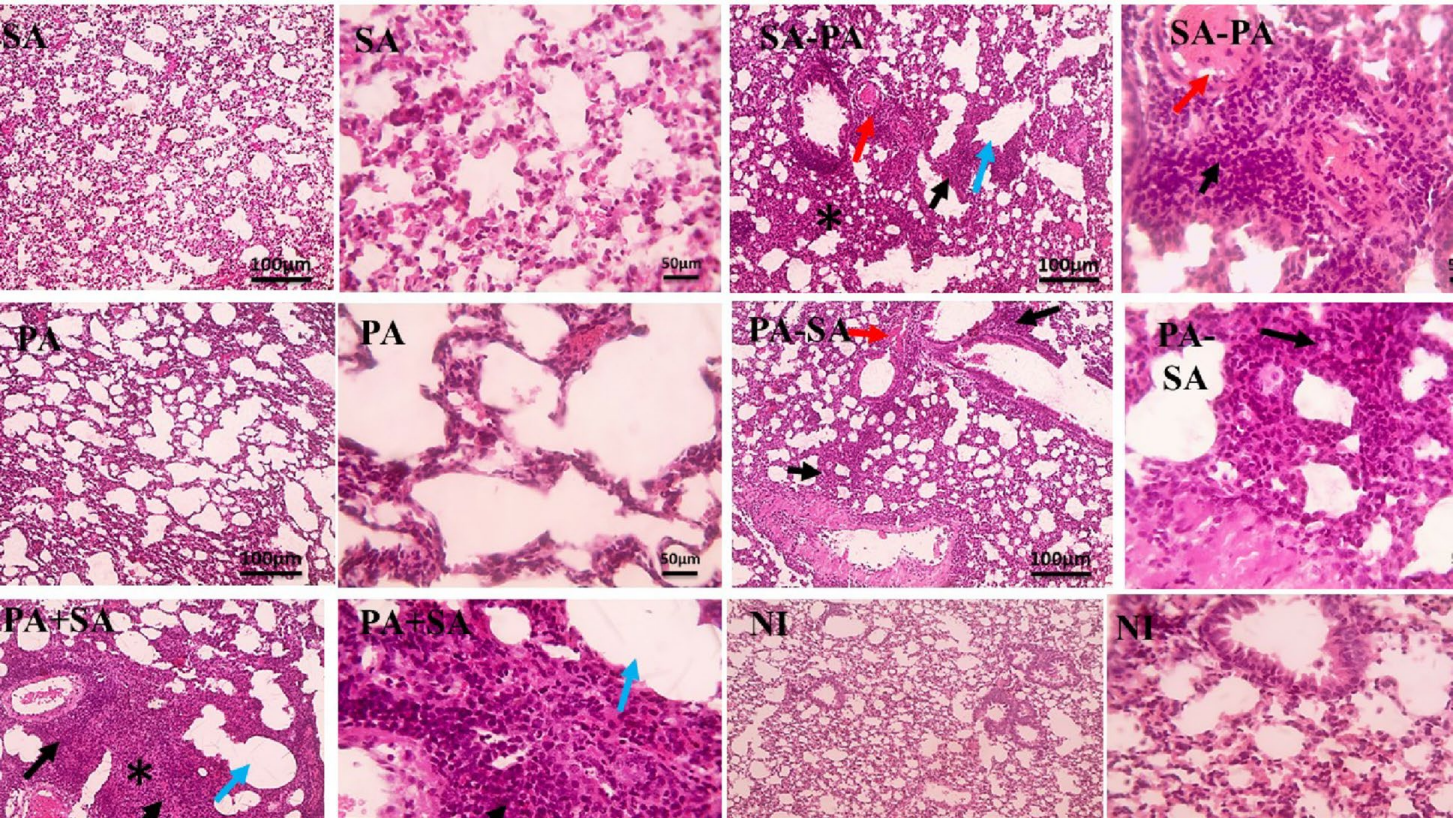
Microscopic pictures of Hematoxylin-eosin-stained lungs sections of different groups of mice. PA and SA group: Means mice got infection with *P. aeruginosa* PAO1 strain and *S. aureus* Newman strain, respectively, NI group: Non-infected mice that got sterile PBS only, PA-SA group: Mice infected with *P. aeruginosa* PAO1 strain followed *S. aureus* Newman strain three days a part, SA-PA group: Mice infected with *S. aureus* Newman strain followed by *P. aeruginosa* PAO1 strain three days apart, PA + SA group: Mice infected with *P. aeruginosa* PAO1 strain and *S. aureus* Newman strain simultaneously. Red arrows: lung congestion, blue arrow: alveolar emphysema, thin black arrow: perivascular and peribronchiolar inflammation and (*): alveolar collapse (narrowing of the alveolar lumen)

The histology of the lungs was evaluated after 7 days of infection by either single or both bacteria species. Unfortunately, due to the unexpected mortality of most experimental animals before tissue collection, histology could only be conducted on one representative mouse. It should be mentioned that, the purpose of histology was for qualitative supportive observation rather than a quantitative comparison. Microscopic pictures of Hematoxylin-eosin-stained lungs sections showed no structural damage, inflammation or congestion in control groups PA, SA and NI. The SA-PA group showed the most obvious change, where severe perivascular and peribronchiolar inflammation mainly neutrophils infiltration, marked thickening of alveolar wall, congestion, alveolar emphysema and collapse were observed (Fig. 6). On the other side, PA-SA and PA + SA groups showed mild histological pattern (Fig. 6).

## Discussion

Different studies have reported that polymicrobial infections produce severe infections than single ones [11]. Several studies have shown that *S. aureus*/*P. aeruginosa* co-infections are one of the most common polymicrobial infections [4, 11, 40, 41]. Most of these studies, have illustrated the impact of *P. aeruginosa* on *S. aureus* antibiotic resistance and pathogenicity [4, 11, 40]. The main aim of this study was to investigate the impact of co-cultures of *P. aeruginosa* and *S. aureus* on antibiotic resistance, virulence and biofilm formation in *P. aeruginosa.*

Initially, 16 co-culture mixtures of *S. aureus*/*P. aeruginosa* were designed (Table 2). These mixtures were examined, via growth competition assay, for the type growth interaction (co-existence or competition). The competition assay showed that 14 mixtures of *S. aureus*/*P. aeruginosa* were able to co-exist. These mixtures were further used in this work to investigate the impact of *S. aureus*/*P. aeruginosa* on antibiotic resistance and virulence of *P. aeruginosa*.

It has been shown that different stages of growth and environmental conditions, including media and planktonic versus biofilm modes of growth, can promote the synergistic interaction of *P. aeruginosa* and *S. aureus* [42, 43]. The Ps5/Sr7 and Ps5/Ss8 mixtures showed a competitive type of interaction. This may be a result of the secretion of proteinaceous toxins through the *S. aureus* type VII secretion system (T7SS) that inhibit the growth of other bacterial specie [44]. It was also reported that *S. aureus* can bio-transform pyochelin, a siderophore produced by *P. aeruginosa*, thereby reducing its availability and hindering *P. aeruginosa* iron acquisition [45].

Several reports have highlighted that the presence of two or more species alters the susceptibility of individual bacterial species through different mechanisms including; physiological or transcriptional alterations, or a combination of both [24, 46]. Our results revealed that, *P. aeruginosa* resistance to cefepime, ceftazidime, gentamicin, ciprofloxacin and meropenem antibiotics increased by 78.6%, 85.7%, 78.6%, 71.4% and 92.9%, respectively in co-culture mixtures compared to *P. aeruginosa* mono-cultures (Fig. 1). It was reported that *P. aeruginosa* resistance in co-culture with *S. aureus* is attributed to interaction between *S. aureus* protein A (SpA) and *P. aeruginosa* Psl [40, 47]. Interestingly, all the naturally co-isolated mixtures showed a significant increase in the survival rate of *P. aeruginosa* in co-culture except for the mixture P8/S9 in the case of cefepime antibiotic.

Co-culture of *P. aeruginosa*/*S. aureus* was reported to affect the expression of virulence encoding genes in *P. aeruginosa* [48]. Our results revealed that the production of all the examined virulence factors increased significantly in most of the *P. aeruginosa* isolates in the investigated co-cultures except for gelatinase production (which only increased in 3 co-cultures mixtures) (Fig. 2). Interestingly, the PAO1/Newman co-culture mixture was associated with significant increase in all of the considered virulence factors of *P. aeruginosa* PAO1 strain. Moreover, all the natural co-isolated mixtures showed significant increase in the production of elastase and protease for *P. aeruginosa* strains. Supporting our results, several studies have reported that in polymicrobial infections, *S. aureus* increases the virulence factors (pyocyanin, pyoverdine, alginate and biofilm) of *P. aeruginosa* [40, 41, 49, 50]. On the other side, previous study showed a significant inhibitory effect of *S. aureus* on *P. aeruginosa* virulence factors (phenazine and pyochelin) in the case of co-culture [51].

Our study demonstrated that, the biofilm-forming abilities were enhanced after co-culture of *S. aureus*/ *P. aeruginosa*, in comparison with the cumulative forming ability of their individual mono-cultures, in 9 mixtures, while 4 mixtures were not changed and one mixture was shifted to a lower category of biofilm formation (Table S4 Supplementary material). Interestingly, the two naturally co-isolated mixtures; P8/S9 and P10/S11 showed the highest increase in biofilm formation by 522% and 480%, respectively (Fig. 3a). It was reported that cultivation of both bacteria leads to the formation of a more rigid biofilm, where *S. aureus* is located mainly in the upper layers, while *P. aeruginosa* can be found mostly in the lower layers of the biofilm [15]. Additionally, it was indicated that cultivation of *P. aeruginosa* with *S. aureus* produced a more rapid biofilm through the formation of large bacterial aggregates that exceeded the amount in mono-culture biofilm, illustrating the high biofilm formation activity among the naturally co-existed mixtures [5].

Co-culture of *P. aeruginosa* and *S. aureus* increased the expression of *P. aeruginosa* virulence encoding genes, where 92.9%, 100% and 92.9% of the co-culture mixtures significantly up-regulated *pelA*, *lasB* and *lasA* genes, respectively compared to their corresponding *P. aeruginosa* mono-cultures (Fig. 4). Up-regulation of virulence genes in *P. aeruginosa* may be a result of an adaptive strategy that *P. aeruginosa* uses to enhance its competitive ability against *S. aureus*, where the elevated gene expressions promote aggressive behaviors, such as increase protease or biofilm producing activities that enable *P. aeruginosa* to lyse competing bacteria and access essential nutrients like iron [41].

Previous studies have demonstrated that microorganisms modulate their gene expression and lifestyle to adapt to competitive niches. For instance, it was reported that in most co-culture conditions the QS systems of *P. aeruginosa* specifically the *lasI*/*lasR* operon and its dependent virulence factors including; *lasA*, *lasB* and biofilm-associated genes, were significantly up-regulated with the exception of one mixture [41]. On the other hand, the anti-staphylococcal product (LasA protease) was not up-regulated under their experimental conditions in *P. aeruginosa* [51].

To mimic the co-infection of *P. aeruginosa*/ *S. aureus* in human we performed in vivo studies in animal to study the effect of co-infection of both bacteria species on the pathogenicity of *P. aeruginosa.* Our results revealed that co-infecting with *P. aeruginosa* PAO1 and *S. aureus* Newman simultaneously has the highest rate of mortality. Infecting the mice with the two bacteria species three days a part leads to the same rate of mortality, however infection with *S. aureus* first, then *P. aeruginosa* accelerates mice death in comparison with the PA-SA group (Fig. 5a). Several animal models have been proposed for studying *S. aureus* or *P. aeruginosa* pathogenicity [52]. Moreover, previous studies reported that the success of *S. aureus* colonization during co-infection was correlated with the extent of *P. aeruginosa* colonization in the lungs, irrespective of the interaction type between the two pathogens [35]. On the other hand, it was demonstrated that, pre-infection with *S. aureus* decreased the mortality rate caused by *P. aeruginosa*, where *P. aeruginosa* and *S. aureus* co-existed in the mouse lungs without interfering with each other [34].

Regarding histological examination of the lung, our results revealed that mice groups that were infected by either one of the two pathogens showed no inflammation. The SA-PA group showed the most severe perivascular and peribronchiolar inflammation. Moreover, both PA-SA and PA + SA groups were associated with mild inflammation in the lung (Fig. 6). Our results were in agreement with that previously reported, where *S. aureus* pre-colonization represented a risk factor for initial *P. aeruginosa* airway infection [34].

## Conclusions

The study emphasizes the significant impact of the co-existence of *S. aureus*/ *P. aeruginosa* on *P. aeruginosa* antibiotic resistance and virulence production. Co-culturing with *S. aureus* led to an increase in *P. aeruginosa* resistance to multiple antimicrobial classes, such as cefepime, ceftazidime, gentamicin, ciprofloxacin and meropenem. Moreover, there was a notable increase in the production of key virulence factors, including pyocyanin, elastase, protease, hemolysin, lecithinase, gelatinase, and biofilm formation. Moreover, gene expression analysis confirmed the up-regulation of virulence-related genes (*pelA*, *lasB*, and *lasA*) in co-cultured isolates. The in vivo mouse infection model supported these findings, showing higher mortality rate, increase in bacterial loads in the lungs, and heightened lung inflammation in co-infected groups. These findings underscore the potential for interspecies interactions to enhance *P. aeruginosa* pathogenicity and antibiotic resistance, underscoring the clinical importance of polymicrobial infections and the necessity of considering microbial co-existence in antimicrobial therapy strategies.

## Supplementary Information

Below is the link to the electronic supplementary material.

Supplementary Material 1

## Data Availability

No datasets were generated or analysed during the current study.

## Abbreviations

*P. aeruginosa*: *Pseudomonas aeruginosa*
*S. aureus*: *Staphylococcus aureus*
CF: Cystic fibrosis
QS: Quorum sensing
CLSI: Clinical Laboratory Standard Institute
CA: Cetrimide agar
MSA: Mannitol salt agar
FEP: Cefepime
CAZ: Ceftazidime
CN: Gentamicin
CIP: Ciprofloxacin
MEM: Meropenem
ATM: Aztreonam
AZM: Azithromycin
DO: Doxycycline
TSB: Tryptic soy broth
CFU: Colony-forming unit
FC: Fold of change
MHB: Muller Hinton broth
MIC: Minimum inhibitory concentration
LB broth: Luria-Bertani broth
OD_T_: The mean optical density of the solubilized biofilm
ODc: Cut-off OD
SD: Standard deviation
RT-PCR: Real-time polymerase chain reaction
PBS: Phosphate buffered saline
T7SS: Type VII secretion system

## References

[1] Anju VT, Busi S, Imchen M, Kumavath R, Mohan MS, Salim SA et al. Polymicrobial infections and biofilms: clinical significance and eradication strategies. Antibiot (Basel). 2022;11(12):1731. 10.3390/antibiotics11121731} 10.3390/antibiotics11121731 PMC9774821 36551388

[2] Nickol ME, Ciric J, Falcinelli SD, Chertow DS, Kindrachuk J. Characterization of host and bacterial contributions to lung barrier dysfunction following co-infection with 2009 pandemic influenza and methicillin resistant* Staphylococcus aureus*. Viruses. 2019;11(2):116. 10.3390/v11020116} 10.3390/v11020116 PMC6409999 30699912

[3] Tahmasebi H, Dehbashi S, Arabestani MR. Co-harboring of mcr-1 and β-lactamase genes in Pseudomonas aeruginosa by high-resolution melting curve analysis (HRMA): molecular typing of Superbug strains in bloodstream infections (BSI). Infect Genet Evol. 2020;85:104518. 32891877 10.1016/j.meegid.2020.104518

[4] Bernardy EE, Raghuram V, Goldberg JB. Staphylococcus aureus and Pseudomonas aeruginosa isolates from the same cystic fibrosis respiratory sample coexist in coculture. Microbiol Spectr. 2022;10(4):e0097622. 35867391 10.1128/spectrum.00976-22 PMC9431432

[5] Vestweber PK, Wächter J, Planz V, Jung N, Windbergs M. The interplay of Pseudomonas aeruginosa and Staphylococcus aureus in dual-species biofilms impacts development, antibiotic resistance and virulence of biofilms in in vitro wound infection models. PLoS ONE. 2024;19(5):e0304491. 38805522 10.1371/journal.pone.0304491 PMC11132468

[6] Liao C, Huang X, Wang Q, Yao D, Lu W. Virulence factors of Pseudomonas aeruginosa and antivirulence strategies to combat its drug resistance. Front Cell Infect Microbiol. 2022;12:926758. 35873152 10.3389/fcimb.2022.926758 PMC9299443

[7] Jurado-Martín I, Sainz-Mejías M, McClean S. Pseudomonas aeruginosa: an audacious pathogen with an adaptable arsenal of virulence factors. Int J Mol Sci. 2021;22(6):3128. } 10.3390/ijms22063128} 10.3390/ijms22063128 PMC8003266 33803907

[8] Sharma S, Mohler J, Mahajan SD, Schwartz SA, Bruggemann L, Aalinkeel R. Microbial biofilm: a review on formation, infection, antibiotic resistance, control measures, and innovative treatment. Microorganisms. 2023;11(6):1614. 10.3390/microorganisms11061614} 10.3390/microorganisms11061614 PMC10305407 37375116

[9] Shahbandeh M, Moosazadeh Moghaddam M, Golmohammadi R, Mirnejad R. The antimicrobial effect of quorum sensing autoinducers of Pseudomonas aeruginosa, C12-HSL and C4-HSL, against MDR Staphylococcus aureus isolates. Comp Immunol Microbiol Infect Dis. 2022;81:101747. 35030534 10.1016/j.cimid.2022.101747

[10] Ibberson CB, Barraza JP, Holmes AL, Cao P, Whiteley M. Precise Spatial structure impacts antimicrobial susceptibility of S. aureus in polymicrobial wound infections. Proc Natl Acad Sci U S A. 2022;119(51):e2212340119. 36520668 10.1073/pnas.2212340119 PMC9907066

[11] Briaud P, Bastien S, Camus L, Boyadjian M, Reix P, Mainguy C, et al. Impact of coexistence phenotype between Staphylococcus aureus and Pseudomonas aeruginosa isolates on clinical outcomes among cystic fibrosis patients. Front Cell Infect Microbiol. 2020;10:266. 32582568 10.3389/fcimb.2020.00266 PMC7285626

[12] Ezeador C, Ejikeugwu C, Ushie S, Agbakoba N, Isolation. Identification and prevalence of* Pseudomonas aeruginosa* isolates from clinical and environmental sources in Onitsha Metropolis, Anambra state. Eur J Med Health Sci. 2020;2(2). } 10.24018/ejmed.2020.2.2.188}

[13] Bauer AW, Kirby WM, Sherris JC, Turck M. Antibiotic susceptibility testing by a standardized single disk method. Am J Clin Pathol. 1966;45(4):493–6. 5325707

[14] CLSI. Performance standards for antimicrobial susceptibility testing. Clinical and laboratory standard institute. 2021.

[15] Trizna E, Yarullina M, Baidamshina D, Mironova A, Akhatova F, Rozhina E, et al. Bidirectional alterations in antibiotics susceptibility in Staphylococcus aureus-Pseudomonas aeruginosa dual-species biofilm. Sci Rep. 2020;10:14849. 32908166 10.1038/s41598-020-71834-w PMC7481796

[16] Alford MA, Mann S, Akhoundsadegh N, Hancock REW. Competition between Pseudomonas aeruginosa and Staphylococcus aureus is dependent on intercellular signaling and regulated by the NtrBC two-component system. Sci Rep. 2022;12(1):9027. 35637237 10.1038/s41598-022-12650-2 PMC9150766

[17] Briaud P, Camus L, Bastien S, Doléans-Jordheim A, Vandenesch F, Moreau K. Coexistence with Pseudomonas aeruginosa alters Staphylococcus aureus transcriptome, antibiotic resistance and internalization into epithelial cells. Sci Rep. 2019;9(1):16564. 31719577 10.1038/s41598-019-52975-z PMC6851120

[18] Essar DW, Eberly L, Hadero A, Crawford IP. Identification and characterization of genes for a second anthranilate synthase in Pseudomonas aeruginosa: interchangeability of the two anthranilate synthases and evolutionary implications. J Bacteriol. 1990;172(2):884–900. 2153661 10.1128/jb.172.2.884-900.1990 PMC208517

[19] Musthafa KS, Saroja V, Pandian SK, Ravi AV. Antipathogenic potential of marine Bacillus sp. SS4 on N-acyl-homoserine-lactone-mediated virulence factors production in Pseudomonas aeruginosa (PAO1). J Biosci. 2011;36(1):55–67. 21451248 10.1007/s12038-011-9011-7

[20] El-Mowafy SA, Abd El Galil KH, El-Messery SM, Shaaban MI. Aspirin is an efficient inhibitor of quorum sensing, virulence and toxins in Pseudomonas aeruginosa. Microb Pathog. 2014;74:25–32. 25088031 10.1016/j.micpath.2014.07.008

[21] Sato T, Kamaguchi A, Nakazawa F. Purification and characterization of hemolysin from prevotella Oris. J Oral Biosci. 2012;54(2):113–8.

[22] Hassan S, Eid D. Effect of tyrosol on Staphylococcus aureus antimicrobial susceptibility, biofilm formation and virulence factors. Afr J Microbiol Res. 2016;10:687–93.

[23] Lopes Mde F, Simões AP, Tenreiro R, Marques JJ, Crespo MT. Activity and expression of a virulence factor, gelatinase, in dairy enterococci. Int J Food Microbiol. 2006;112(3):208–14. 17046092 10.1016/j.ijfoodmicro.2006.09.004

[24] Abbott C, Grout E, Morris T, Brown HL. Cutibacterium acnes biofilm forming clinical isolates modify the formation and structure of Staphylococcus aureus biofilms, increasing their susceptibility to antibiotics. Anaerobe. 2022;76:102580. 35598875 10.1016/j.anaerobe.2022.102580

[25] Soliman M, Said HS, El-Mowafy M, Barwa R, Novel. PCR detection of CRISPR/Cas systems in Pseudomonas aeruginosa and its correlation with antibiotic resistance. Appl Microbiol Biotechnol. 2022;106(21):7223–34. 36178514 10.1007/s00253-022-12144-1 PMC9592639

[26] Limoli DH, Whitfield GB, Kitao T, Ivey ML, Davis MR Jr., Grahl N et al.* Pseudomonas aeruginosa* alginate overproduction promotes coexistence with* Staphylococcus aureus* in a model of cystic fibrosis respiratory infection. mBio. 2017;8(2):e00186-17. 10.1128/mBio.00186-17} 10.1128/mBio.00186-17 PMC5362032 28325763

[27] Livak KJ, Schmittgen TD. Analysis of relative gene expression data using real-time quantitative PCR and the 2(-Delta delta C(T)) method. Methods. 2001;25(4):402–8. 11846609 10.1006/meth.2001.1262

[28] El-Shaer S, Shaaban M, Barwa R, Hassan R. Control of quorum sensing and virulence factors of Pseudomonas aeruginosa using phenylalanine arginyl β-naphthylamide. J Med Microbiol. 2016;65(10):1194–204. 27498852 10.1099/jmm.0.000327

[29] Meskini M, Ghorbani M, Bahadoran H, zaree A, Esmaeili D. ZOUSH ointment with the properties of antibacterial Moreover, burn wound healing. Int J Pept Res Ther. 2020;26(1):349–55.

[30] Li K, Xu W, Guo Q, Jiang Z, Wang P, Yue Y, et al. Differential macrophage polarization in male and female BALB/c mice infected with coxsackievirus B3 defines susceptibility to viral myocarditis. Circ Res. 2009;105(4):353–64. 19608981 10.1161/CIRCRESAHA.109.195230

[31] Alonaizan R, Woods S, Hargrave KE, Roberts CW. An exaggerated immune response in female BALB/c mice controls initial* Toxoplasma gondii* multiplication but increases mortality and morbidity relative to male mice. Pathogens. 2021;10(9):1154. 10.3390/pathogens10091154} 10.3390/pathogens10091154 PMC8470933 34578186

[32] Patel S, Patel S, Kotadiya A, Patel S, Shrimali B, Tank M, et al. Comparative analysis of the effect of sex and age on the hematological and biochemical profile of BALB/c and C57BL/6 inbred mice. J Am Assoc Lab Anim Sci. 2025;64(1):132–45. 40035280 10.30802/AALAS-JAALAS-24-075 PMC11808370

[33] Ferguson LT, Rashied AA, Liang Z, Yumoto T, Anyalebechi JC, Swift DA, et al. A novel scoring system for humane endpoints in mice with cecal ligation and Puncture-Induced sepsis. Comp Med. 2023;73(6):446–60. 38217069 10.30802/AALAS-CM-22-000124 PMC10752367

[34] Cigana C, Bianconi I, Baldan R, De Simone M, Riva C, Sipione B, et al. Staphylococcus aureus impacts Pseudomonas aeruginosa chronic respiratory disease in murine models. J Infect Dis. 2018;217(6):933–42. 29216403 10.1093/infdis/jix621

[35] Millette G, Langlois JP, Brouillette E, Frost EH, Cantin AM, Malouin F. Despite antagonism in vitro, Pseudomonas aeruginosa enhances Staphylococcus aureus colonization in a murine lung infection model. Front Microbiol. 2019;10:2880. 31921058 10.3389/fmicb.2019.02880 PMC6923662

[36] Peres-Emidio EC, Freitas GJC, Costa MC, Gouveia-Eufrasio L, Silva LMV, Santos APN, et al. Pseudomonas aeruginosa infection modulates the immune response and increases mice resistance to Cryptococcus Gattii. Front Cell Infect Microbiol. 2022;12:811474. 35548467 10.3389/fcimb.2022.811474 PMC9083911

[37] Shrum B, Anantha RV, Xu SX, Donnelly M, Haeryfar SM, McCormick JK, et al. A robust scoring system to evaluate sepsis severity in an animal model. BMC Res Notes. 2014;7:233. 24725742 10.1186/1756-0500-7-233 PMC4022086

[38] Dudis RS, Wong TY, Escatte MG, Alamneh YA, Abu-Taleb R, Su W, et al. Longitudinal temperature measurement can determine humane endpoints in BALB/c mouse models of ESKAPEE infection. Virulence. 2023;14(1):2186331. 36976806 10.1080/21505594.2023.2186331 PMC10054282

[39] Ainsworth S, Ketter PM, Yu JJ, Grimm RC, May HC, Cap AP, et al. Vaccination with a live attenuated acinetobacter baumannii deficient in thioredoxin provides protection against systemic acinetobacter infection. Vaccine. 2017;35(26):3387–94. 28522011 10.1016/j.vaccine.2017.05.017 PMC5510955

[40] Armbruster CR, Wolter DJ, Mishra M, Hayden HS, Radey MC, Merrihew G et al.* Staphylococcus aureus* protein A mediates interspecies interactions at the cell surface of* Pseudomonas aeruginosa*. mBio. 2016;7(3):e00538-16. 10.1128/mBio.00538-16} 10.1128/mBio.00538-16 PMC4895107 27222468

[41] Dehbashi S, Alikhani MY, Tahmasebi H, Arabestani MR. The inhibitory effects of Staphylococcus aureus on the antibiotic susceptibility and virulence factors of Pseudomonas aeruginosa: A549 cell line model. AMB Express. 2021;11(1):50. 33786713 10.1186/s13568-021-01210-y PMC8010066

[42] Cendra MDM, Blanco-Cabra N, Pedraz L, Torrents E. Optimal environmental and culture conditions allow the in vitro coexistence of Pseudomonas aeruginosa and Staphylococcus aureus in stable biofilms. Sci Rep. 2019;9(1):16284. 31705015 10.1038/s41598-019-52726-0 PMC6841682

[43] Barraza JP, Whiteley M. A* Pseudomonas aeruginosa* antimicrobial affects the biogeography but not fitness of* Staphylococcus aureus* during coculture. mBio. 2021;12(2):e00047-21. 10.1128/mBio.00047-21} 10.1128/mBio.00047-21 PMC8092195 33785630

[44] Luo H, Ni L, Chen T, Huang L, Zhang X, Li X, et al. Intraspecific Cooperation allows the survival of Staphylococcus aureus staff: a novel strategy for disease relapse. BMC Infect Dis. 2024;24(1):1092. 39354412 10.1186/s12879-024-10001-2 PMC11445958

[45] Jenul C, Keim K, Jens J, Zeiler M, Schilcher K, Schurr M, et al. Pyochelin biotransformation by Staphylococcusaureus shapes bacterial competition with Pseudomonas aeruginosa in polymicrobial infections. Cell Rep. 2023;42:112540. 37227819 10.1016/j.celrep.2023.112540 PMC10592502

[46] Kalan LR, Meisel JS, Loesche MA, Horwinski J, Soaita I, Chen X, et al. Strain-and species-level variation in the Microbiome of diabetic wounds is associated with clinical outcomes and therapeutic efficacy. Cell Host Microbe. 2019;25(5):641–55. e5. 31006638 10.1016/j.chom.2019.03.006 PMC6526540

[47] Beaudoin T, Yau YCW, Stapleton PJ, Gong Y, Wang PW, Guttman DS, et al. Staphylococcus aureus interaction with Pseudomonas aeruginosa biofilm enhances tobramycin resistance. NPJ Biofilms Microbiomes. 2017;3:25. 29062489 10.1038/s41522-017-0035-0 PMC5648753

[48] Biswas L, Götz F. Molecular mechanisms of Staphylococcus and Pseudomonas interactions in cystic fibrosis. Front Cell Infect Microbiol. 2021;11:824042. 35071057 10.3389/fcimb.2021.824042 PMC8770549

[49] Hotterbeekx A, Kumar-Singh S, Goossens H, Malhotra-Kumar S. In vivo and in vitro interactions between Pseudomonas aeruginosa and Staphylococcus spp. Front Cell Infect Microbiol. 2017;7:106. 28421166 10.3389/fcimb.2017.00106 PMC5376567

[50] Alves PM, Al-Badi E, Withycombe C, Jones PM, Purdy KJ, Maddocks SE. Interaction between* Staphylococcus aureus* and* Pseudomonas aeruginosa* is beneficial for colonisation and pathogenicity in a mixed biofilm. Pathog Dis. 2018;76(1):10.1093/femspd/fty003. } } 10.1093/femspd/fty003} 10.1093/femspd/fty003 29342260

[51] Tognon M, Köhler T, Luscher A, van Delden C. Transcriptional profiling of Pseudomonas aeruginosa and Staphylococcus aureus during in vitro co-culture. BMC Genomics. 2019;20(1):30. 30630428 10.1186/s12864-018-5398-y PMC6327441

[52] Silva-Santana G, Aguiar-Alves F, Lenzi-Almeida KC, Lopes VGS, Silva LE, Hirata Júnior R, et al. Pathological profiles of systemic infections by Panton-Valentine leukocidin-positive, methicillin-resistant Staphylococcus aureus strains in a murine model. J Appl Microbiol. 2020;128(6):1820–42. 31999872 10.1111/jam.14598

