# Supplementary material for: Coexistence with Staphylococcus aureus modulates the virulence and antibiotic resistance of Pseudomonas aeruginosa
Source: Ann Clin Microbiol Antimicrob. 2026 Jan 28;25:14. doi: 10.1186/s12941-025-00843-2 (PMC12922201; doi:10.1186/s12941-025-00843-2)
Supplement: Supplementary file 1 — Supplementary Material 1 [file 12941_2025_843_MOESM1_ESM.docx]

**Table 1 supplementary material: *P. aeruginosa* and *S. aureus* isolates collected in this study.**

| ***P. aeruginosa* isolates** | | | | ***S. aureus* isolates** | | | |
| --- | --- | --- | --- | --- | --- | --- | --- |
| **Isolate code** | **Clinical source** | **Isolate code** | **Clinical source** | **Isolate code** | **Clinical source** | **Isolate code** | **Clinical source** |
| P1 | Sputum | P16 | Sputum | S1 | Wound | S20 | Sputum |
| P2 | Wound | P17 | Sputum | S2 | Wound | S21 | Sputum |
| P3 | Wound | P18 | Sputum | S3 | Wound | S22 | Sputum |
| P4 | Pus | P19 | Wound | S4 | Sputum | S23 | Sputum |
| P5 | Pus | P20 | Pus | S5 | Wound | S24 | Sputum |
| P6 | Pus | P21 | Wound | S6 | Wound | S25 | Sputum |
| P7 | Wound | P22 | Sputum | S7 | Pus | S26 | Sputum |
| P8 | Pus | P23 | Sputum | S8 | Pus | S27 | Sputum |
| P9 | Wound | P24 | Sputum | S9 | Pus | S28 | Sputum |
| P10 | Pus | P25 | Sputum | S10 | Wound | S29 | Pus |
| P11 | Wound | P26 | Sputum | S11 | Pus | S30 | Pus |
| P12 | Wound | P27 | Wound | S12 | Pus | S31 | Pus |
| P13 | Diabetic foot | P28 | Wound | S13 | Diabetic foot | S32 | Diabetic foot |
| P14 | Pus | P29 | Wound | S14 | Pus | S33 | Pus |
| P15 | Sputum |  | | S15 | Wound | S34 | Pus |
|  | | | | S16 | Wound | S35 | Pus |
|  |  |  |  | S17 | Wound | S36 | Pus |
|  |  |  |  | S18 | Wound | S37 | Pus |
|  |  |  |  | S19 | Wound | S38 | Pus |

**Table 2 supplementary material: Antibiotic sensitivity patterns of *P. aeruginosa* isolates.**

| **Isolate code** | **FEP** | **AMC** | **CAZ** | **ATM** | **MEM** | **CN** | **CIP** |
| --- | --- | --- | --- | --- | --- | --- | --- |
| P1 | R | R | R | R | R | R | R |
| P2 | R | R | R | R | R | R | R |
| P3 | S | I | S | S | S | S | S |
| P4 | S | I | S | S | S | S | S |
| P5 | S | I | S | S | S | S | S |
| P6 | R | R | R | R | R | R | R |
| P7 | R | R | R | R | R | R | R |
| P8 | S | R | I | S | S | S | S |
| P9 | I | R | S | S | S | R | S |
| P10 | I | I | S | R | S | R | I |
| P11 | S | R | R | S | S | R | R |
| P12 | S | R | R | S | S | R | R |
| P13 | S | R | R | S | S | R | R |
| P14 | R | R | R | R | S | S | R |
| P15 | S | R | R | S | R | R | R |
| P16 | S | R | R | R | S | R | R |
| P17 | S | R | R | S | S | R | I |
| P18 | S | R | R | R | S | S | S |
| P19 | I | R | R | S | S | R | R |
| P20 | I | R | R | S | S | R | R |
| P21 | S | R | R | S | S | S | S |
| P22 | S | R | R | S | R | S | S |
| P23 | S | R | R | S | S | R | S |
| P24 | S | R | R | S | R | S | S |
| P25 | S | R | R | S | S | R | S |
| P26 | S | R | R | S | S | S | R |
| P27 | S | R | R | R | R | S | S |
| P28 | S | R | R | S | R | S | S |
| P29 | S | R | R | S | S | R | S |

**FEP:** Cefepime, **AMC:** Ampicillin/clavulanic acid, **CAZ:** Ceftazidime, **ATM:** Aztreonam, **MEM:** Meropenem, **CN:** Gentamicin, **CIP:** Ciprofloxacin, **S:** Sensitive, **R:** Resistant, **I:** intermediate.

**Table 3 supplementary material: Antibiotic sensitivity patterns of *S. aureus* isolates.**

| **Isolate code** | **FEP** | **AMC** | **CN** | **CIP** | **DO** | **AZM** |
| --- | --- | --- | --- | --- | --- | --- |
| **S1** | S | S | S | S | S | I |
| **S2** | S | S | S | S | S | S |
| **S3** | S | S | S | S | I | S |
| **S4** | R | R | R | R | R | R |
| **S5** | R | R | R | R | R | I |
| **S6** | R | R | R | R | I | R |
| **S7** | R | R | R | I | R | R |
| **S8** | S | S | S | S | S | I |
| **S9** | S | R | S | S | I | S |
| **S10** | S | R | S | S | S | S |
| **S11** | S | R | R | R | R | S |
| **S12** | S | R | R | R | R | S |
| **S13** | S | R | R | R | R | S |
| **S14** | S | R | R | R | R | S |
| **S15** | S | R | S | S | I | S |
| **S16** | S | R | R | S | R | S |
| **S17** | S | R | S | S | I | S |
| **S18** | S | R | S | S | S | S |
| **S19** | S | R | S | S | I | S |
| **S20** | S | R | R | I | R | S |
| **S21** | S | S | S | R | R | R |
| **S22** | S | R | R | S | R | S |
| **S23** | S | R | S | S | I | S |
| **S24** | S | R | S | S | I | S |
| **S 25** | S | R | S | R | S | R |
| **S26** | S | R | S | R | S | S |
| **S27** | S | R | S | S | R | R |
| **S28** | S | R | S | S | S | R |
| **S29** | S | R | R | S | S | R |
| **S30** | S | S | S | R | R | S |
| **S31** | S | S | R | R | S | I |
| **S32** | S | S | R | S | R | R |
| **S33** | S | R | S | S | R | R |
| **S34** | S | R | R | S | S | R |
| **S35** | S | R | S | S | S | R |
| **S36** | R | R | S | R | S | R |
| **S37** | S | R | S | R | S | R |
| **S38** | S | R | R | S | R | S |

**FEP:** Cefepime, **AMC:** Ampicillin/clavulanic acid, **CN:** Gentamicin, **CIP:** Ciprofloxacin, **DO:** Doxycycline, **AZM:** Azithromycin, **S:** Sensitive, **R:** Resistant, **I:** intermediate.

**Table 4 supplementary material: Biofilm production indicated by OD_T_ in co-culture and mixture of individual mono-cultures and different biofilm categories.**

| **Category** | **Co-culture code:** | **OD_T_ of co-culture** | **OD_T_ of mixtures of individual mono-cultures** |
| --- | --- | --- | --- |
| **Standard:** | PAO1/Newman | 0.18 (Moderate-adherent) | 0.13 (Weak-adherent) |
|  | PAO1/Ss2 | 0.31(Moderate-adherent) | 0.14 (Weak-adherent) |
|  | PAO1/Sr4 | 0.29 (Moderate-adherent) | 0.12 (Weak-adherent) |
|  | Ps4/Newman | 0.14 (Weak-adherent) | 0.11 (Weak-adherent) |
|  | Pr7/Newman | 0.24 (Moderate- adherent) | 0.07 (Non-adherent) |
|  | **% of mixtures** | **Weak-adherent: 20%**  **Moderate- adherent: 80%** | **Non-adherent: 20%**  **Weak- adherent: 80%** |
| **Naturally co-isolated:** | P8/S9 | 0.26 (Moderate-adherent) | 0.05 (Non-adherent) |
|  | P9/S10 | 0.17 (Moderate-adherent) | 0.18 (Moderate-adherent) |
|  | P10/S11 | 0.24 (Moderate-adherent) | 0.05 (Non-adherent) |
|  | **% of mixtures** | **Moderate- adherent: 100%** | **Non-adherent: 66.7%**  **Moderate- adherent: 33.3%** |
| **Random designed:** | Pr2/Ss3 | 0.2 (Moderate-adherent) | 0.25 (Moderate-adherent) |
|  | Ps3/Ss3 | 0.2 (Moderate-adherent) | 0.15 (Weakly-adherent) |
|  | Pr1/Sr5 | 0.15 (Weak-adherent) | 0.22 (Moderate-adherent) |
|  | Ps5/Sr5 | 0.44 (Strong-adherent) | 0.17 (Moderate-adherent) |
|  | Pr6/Ss1 | 0.58 (Strong-adherent) | 0.24 (Moderate-adherent) |
|  | Pr6/Sr6 | 0.14 (Weak-adherent) | 0.13 (Weak-adherent) |
|  | **% of mixtures** | **Strong- adherent: 33.3%**  **Moderate- adherent: 33.3%**  **Weak-adherent: 33.3%** | **Moderate- adherent: 66.7%**  **Weak-adherent: 33.3%** |
